# Phase 1 LITESPARK-001 study of belzutifan in participants with advanced solid tumors: Results of the glioblastoma expansion cohort

**DOI:** 10.1093/noajnl/vdaf242

**Published:** 2025-11-20

**Authors:** Roy E Strowd, Macarena de la Fuente, Todd M Bauer, Kyriakos P Papadopoulos, Ke Chen, Rodolfo F Perini, Yanfang Liu, David F McDermott

**Affiliations:** Department of Neurology, Wake Forest University School of Medicine, Winston-Salem (R.S.); Sylvester Comprehensive Cancer Center and Department of Neurology, University of Miami, Miami (M.F.); Greco-Hainsworth Centers for Research/Tennessee Oncology, PLLC, Nashville (T.B.); Hematology/Oncology, START San Antonio, San Antonio (K.P.); Merck & Co., Inc, Rahway (K.C., R.P., Y.L.); Merck & Co., Inc, Rahway (K.C., R.P., Y.L.); Merck & Co., Inc, Rahway (K.C., R.P., Y.L.); Internal Medicine-Medical Oncology, Beth Israel Deaconess Medical Center, Boston (D.M.)

**Keywords:** belzutifan, hypoxia-inducible factor, IDH wild-type glioblastoma, recurrence

## Abstract

Recurrent isocitrate dehydrogenase (IDH) wild-type glioblastoma has no established standard-of-care treatment. Preclinical models have demonstrated that hypoxia-inducible factor-2 alpha (HIF-2α) contributes to stabilizing the hypoxic environment in glioblastoma. It was thus hypothesized that the first-in-class HIF-2α inhibitor, belzutifan, may improve outcomes in patients with recurrent IDH wild-type glioblastoma. The glioblastoma cohort of the phase 1 LITESPARK-001 trial enrolled participants who had received radiotherapy and temozolomide. Although targeting HIF-2α may have a role in advancing treatment options for patients with glioblastoma, single-agent belzutifan did not have antitumor activity in this cohort.

Dysregulation of the cellular hypoxic response is a prominent feature of the tumor microenvironment in glioblastoma and contributes to therapeutic resistance by regulating cell cycle checkpoint expression, autophagy, and antioxidant/redox activity.^1^^,^^2^    These mechanisms promote invasion and cancer cell stemness.^1^ Hypoxia-inducible factor-2 alpha (HIF-2α) has been suggested as a potential therapeutic target due to its role in stabilizing the hypoxic environment in preclinical models of glioblastoma.^3^^,^^4^

The first-in-class oral HIF-2α inhibitor belzutifan was first approved for the treatment of certain patients with von Hippel-Lindau disease–associated renal cell carcinoma (RCC), central nervous system (CNS) hemangioblastomas, or pancreatic neuroendocrine tumors and more recently, for the treatment of patients with advanced RCC following programmed cell death protein/ligand 1 (PD-[L]1) and vascular endothelial growth factor tyrosine kinase inhibitors.^5^^,^^6^ The open-label, first-in-human phase 1 LITESPARK-001 trial evaluated belzutifan monotherapy in advanced solid tumors.^7^ Here, we report results from the LITESPARK-001 glioblastoma expansion cohort.

LITESPARK-001 (NCT02974738) was conducted in two parts: a dose-escalation phase in participants with advanced solid tumors and a dose-expansion phase in participants with specific tumor types.^7^ Study design of the dose-escalation phase has been published.^7^

Participants enrolled in the glioblastoma expansion cohort were aged ≥18 years with histologically confirmed isocitrate dehydrogenase (IDH) wild-type glioblastoma that was first recurrent following radiotherapy and temozolomide per Response Assessment in Neuro-Oncology (RANO) criteria. The study was conducted in accordance with principles of Good Clinical Practice and was approved by the appropriate institutional review boards and regulatory agencies. All participants provided written informed consent before enrollment.

Eligible participants received belzutifan 120 mg orally twice daily 12 hours apart to ensure adequate CNS drug exposure. Tumor imaging was by magnetic resonance imaging with and without gadolinium contrast per current recommendations at baseline, ≤7 days before the week 9 visit, and every 8 weeks thereafter. Tumor response was assessed by the local investigator per RANO criteria. Adverse events (AEs) were collected from the first dose to 28 days after treatment discontinuation and were graded using the National Cancer Institute Common Terminology Criteria for AEs, version 4.03.

End points included objective response rate (ORR; proportion of participants with a complete response or partial response), clinical benefit rate (CBR; proportion of participants with a complete response, partial response, or stable disease), and progression-free survival (PFS; time from first dose to progressive disease [PD] or death from any cause) per RANO criteria by local investigator assessment, and safety. Efficacy was assessed in all participants who received ≥1 dose of study treatment and had a baseline and ≥1 postbaseline disease assessment per RANO criteria, or who had discontinued from the study before their first postbaseline assessment due to death or documented PD. Safety was assessed in all participants who received ≥1 dose of study treatment. ORR and CBR 95% confidence intervals (CIs) were calculated using the two-sided Clopper–Pearson exact binomial method. PFS and associated 95% CIs were estimated using the Kaplan–Meier and Brookmeyer-Crowly methods, respectively. Planned enrollment was 25 participants.

Among 25 enrolled participants, the median age was 63 years (range, 35-75), 60% were male, 84% were White, 92% had a Karnofsky Performance Scale score of ≥70%, and 56% had an unmethylated MGMT status (Supplementary Table 1). The median follow-up (time from first dose to end of study, death, or data cutoff [April 1, 2022]) was 1.9 months (range, 0.7-5.1). Median duration of treatment (time between dates of the first and last doses) was 1.3 months (range, 0.3-3.7). The ORR was 0% (95% CI, 0-14), and the CBR was 8% (95% CI, 1-26; Table 1). At the data cutoff date, 19 of 25 participants (76%) had a PFS event. The median PFS was 1.4 months (95% CI, 1.1-1.8; Table 1 and Supplementary Figure S1). All 25 participants experienced ≥1 all-cause AEs, including 15 participants (60%) who experienced a grade 3-5 AE (grade 5, *n* = 2 [both participants had PD not attributed to study treatment]; Supplementary Table 2). Treatment-related AEs (TRAEs) occurred in 22 participants (88%). Grade 3 or 4 TRAEs occurred in 7 participants (28%). The most common TRAEs were anemia (52%), fatigue (44%), and increased alanine aminotransferase (24%; Supplementary Table 3). TRAEs led to dose interruptions in 2 participants (8%). No participant discontinued treatment or experienced a dose reduction due to TRAEs.

**Table 1.** Best objective response and progression-free survival per response assessment in Neuro-Oncology criteria

	Belzutifan *N* = 25
**Objective response rate^a^, % (95% CI)**	0 (0-14)
**Clinical benefit rate^b^, % (95% CI)**	8 (1-26)
**Best response, *n* (%)**	
Complete response	0 (0)
Partial response	0 (0)
Stable disease	2 (8)
Progressive disease	23 (92)
**Progression-free survival**	
Events, *n* (%)	19 (76)
Median (95% CI), months	1.4 (1.1-1.8)

a Includes participants with complete or partial responses.

b Includes participants with complete response, partial response, or stable disease.

Antitumor activity was not observed with belzutifan monotherapy in participants with recurrent IDH wild-type glioblastoma, and the AE profile of belzutifan was consistent with the known profile of the agent.^5^^,^^6^ Hypoxia contributes to therapeutic resistance in glioblastoma by controlling cellular processes that promote tumorigenesis.^1^^,^^2^ Additionally, hypoxia differentially induces the expression of HIF-2α in glioma stem cells, promotes self-renewal properties of glioma stem cells, and may drive non-stem cells toward a stem-like phenotype.^3^^,^^4^ Lack of antitumor activity in this study suggests that HIF-2α inhibition is not a sole therapeutic target for glioblastoma. Treatment with the anti–PD-1 antibodies pembrolizumab and nivolumab have also failed to improve efficacy in participants with recurrent glioblastoma.^8^^,^^9^ The lack of antitumor activity with belzutifan monotherapy is likely due to the highly heterogeneous and complex glioblastoma tumor microenvironment, which presents a challenge in developing effective single-agent therapies in this disease setting.^1^ Data from preclinical mouse models show that HIF-2α inhibition sensitizes glioblastoma tumors to immune checkpoint inhibition^10^; therefore, combination regimens with an HIF-2α inhibitor and an immune checkpoint inhibitor could be evaluated in patients with glioblastoma. Additionally, innovative approaches such as novel drug designs and novel drug delivery methods could enhance drug penetration of the blood-brain barrier, which constitutes a major obstacle to the effective delivery of current treatments for glioblastoma. Limitations of this study include the small size of the study population and the lack of CNS pharmacokinetic analyses to assess belzutifan penetration of the blood-brain barrier.

Although targeting HIF-2α may have a role in advancing treatment options for patients with glioblastoma, particularly as combinatorial treatment, findings from this study do not support additional studies that target HIF-2α alone as a therapeutic strategy in patients with recurrent IDH wild-type glioblastoma.

## Supplementary Material

vdaf242_Supplementary_Data

## Data Availability

Merck Sharp & Dohme LLC, a subsidiary of Merck & Co., Inc., Rahway, NJ, USA (MSD), is committed to providing qualified scientific researchers access to anonymized data and clinical study reports from the company’s clinical trials for the purpose of conducting legitimate scientific research. MSD is also obligated to protect the rights and privacy of trial participants and, as such, has a procedure in place for evaluating and fulfilling requests for sharing company clinical trial data with qualified external scientific researchers. The MSD data sharing website (available at https://externaldatasharing-msd.com/) outlines the process and requirements for submitting a data request. Applications will be promptly assessed for completeness and policy compliance. Feasible requests will be reviewed by a committee of MSD subject matter experts to assess the scientific validity of the request and the qualifications of the requestors. In line with data privacy legislation, submitters of approved requests must enter into a standard data-sharing agreement with MSD before data access is granted. Data will be made available for request after product approval in the United States and the European Union or after product development is discontinued. There are circumstances that may prevent MSD from sharing requested data, including country or region-specific regulations. If the request is declined, it will be communicated to the investigator. Access to genetic or exploratory biomarker data requires a detailed, hypothesis-driven statistical analysis plan that is collaboratively developed by the requestor and MSD subject matter experts; after approval of the statistical analysis plan and execution of a data-sharing agreement, MSD will either perform the proposed analyses and share the results with the requestor or will construct biomarker covariates and add them to a file with clinical data that is uploaded to an analysis portal so that the requestor can perform the proposed analyses.
