# Supplementary material for: Phase 1 LITESPARK-001 study of belzutifan in participants with advanced solid tumors: Results of the glioblastoma expansion cohort
Source: Neurooncol Adv. 2025 Nov 20;8(1):vdaf242. doi: 10.1093/noajnl/vdaf242 (PMC13123851; doi:10.1093/noajnl/vdaf242)
Supplement: vdaf242_Supplementary_Data [file vdaf242_supplementary_data.zip › Strowd-LS001 Glioblastoma_Supplement.docx]

**Supplementary Material**

**Table of Contents**

**Supplementary Table 1. Participant demographics and baseline characteristics**2

**Supplementary Table 2. Summary of adverse events**4

**Supplementary Table 3. Treatment-related adverse events**5

**Supplementary Figure 1. Kaplan-Meier estimate of progression-free survival per Response Assessment in Neuro-Oncology criteria**7

**Supplementary Table 1. Participant demographics and baseline characteristics**

|  | **Belzutifan**  **N = 25** |
| --- | --- |
| **Age, median (range), years** | 63 (35-75) |
| **Sex, n (%)** |  |
| Male | 15 (60) |
| Female | 10 (40) |
| **Race, n (%)** |  |
| White | 21 (84) |
| Asian | 2 (8) |
| Black or African American | 1 (4) |
| Other | 1 (4) |
| **Ethnicity, n (%)** |  |
| Hispanic or Latino | 5 (20) |
| Not Hispanic or Latino | 20 (80) |
| **Karnofsky Performance Scale score, n (%)** |  |
| 90% or 100% | 11 (44) |
| 70% or 80% | 12 (48) |
| 60% | 2 (8) |
| **MGMT status, n (%)** |  |
| Methylated | 9 (36) |
| Hypermethylated | 1 (4) |
| Unmethylated | 14 (56) |
| Indeterminate | 1 (4) |

MGMT, O6-methylguanine-DNA methyltransferase.

**Supplementary Table 2. Summary of adverse events**

|  | **Belzutifan**  **N = 25** |
| --- | --- |
| **All-cause adverse events, n (%)** |  |
| Any | 25 (100) |
| Grade 3-5 | 15 (60) |
| Serious | 9 (36) |
| Led to treatment discontinuation | 0 (0) |
| Led to dose reduction | 0 (0) |
| Led to dose interruption | 6 (24) |
| Led to death | 2 (8)^a^ |
| **Treatment-related adverse events, n (%)** |  |
| Any | 22 (88) |
| Grade 3 or 4 | 7 (28) |
| Serious | 2 (8) |
| Led to treatment discontinuation | 0 (0) |
| Led to dose reduction | 0 (0) |
| Led to dose interruption | 2 (8) |
| Led to death | 0 (0) |

^a^Both participants died of progressive disease.

**Supplementary Table 3. Treatment-related^a^ adverse events**

| **n (%)** | **Belzutifan**  **N = 25** | |
| --- | --- | --- |
|  | **Any grade** | **Grade 3 or 4^b^** |
| Anemia | 13 (52) | 1 (4) |
| Fatigue | 11 (44) | 2 (8) |
| Alanine aminotransferase increased | 6 (24) | 2 (8) |
| Platelet count decreased | 4 (16) | 0 (0) |
| White blood cell count decreased | 4 (16) | 0 (0) |
| Headache | 3 (12) | 0 (0) |
| Nausea | 3 (12) | 0 (0) |
| Hypoxia | 3 (12) | 2 (8) |
| Hypertension | 3 (12) | 0 (0) |
| Aspartate aminotransferase increased | 2 (8) | 1 (4) |
| Neutrophil count decreased | 2 (8) | 0 (0) |
| Localized edema | 1 (4) | 0 (0) |
| Edema peripheral | 1 (4) | 0 (0) |
| Blood bilirubin increased | 1 (4) | 0 (0) |
| Blood calcium decreased | 1 (4) | 0 (0) |
| Cardiac murmur | 1 (4) | 0 (0) |
| Lymphocyte count decreased | 1 (4) | 0 (0) |
| Memory impairment | 1 (4) | 0 (0) |
| Vomiting | 1 (4) | 0 (0) |
| Fall | 1 (4) | 0 (0) |
| Wound dehiscence | 1 (4) | 1 (4) |
| Decreased appetite | 1 (4) | 0 (0) |
| Hyperglycemia | 1 (4) | 0 (0) |
| Hypoglycemia | 1 (4) | 0 (0) |
| Device-related infection | 1 (4) | 1 (4) |
| Myalgia | 1 (4) | 0 (0) |

Adverse events were coded using Medical Dictionary for Regulatory Activities version 25.0.

^a^Determined by the investigator to be related to the drug.

^b^No Grade 5 treatment-related adverse events occurred.

**Supplementary Figure 1**. Kaplan-Meier estimate of progression-free survival per Response Assessment in Neuro-Oncology criteria


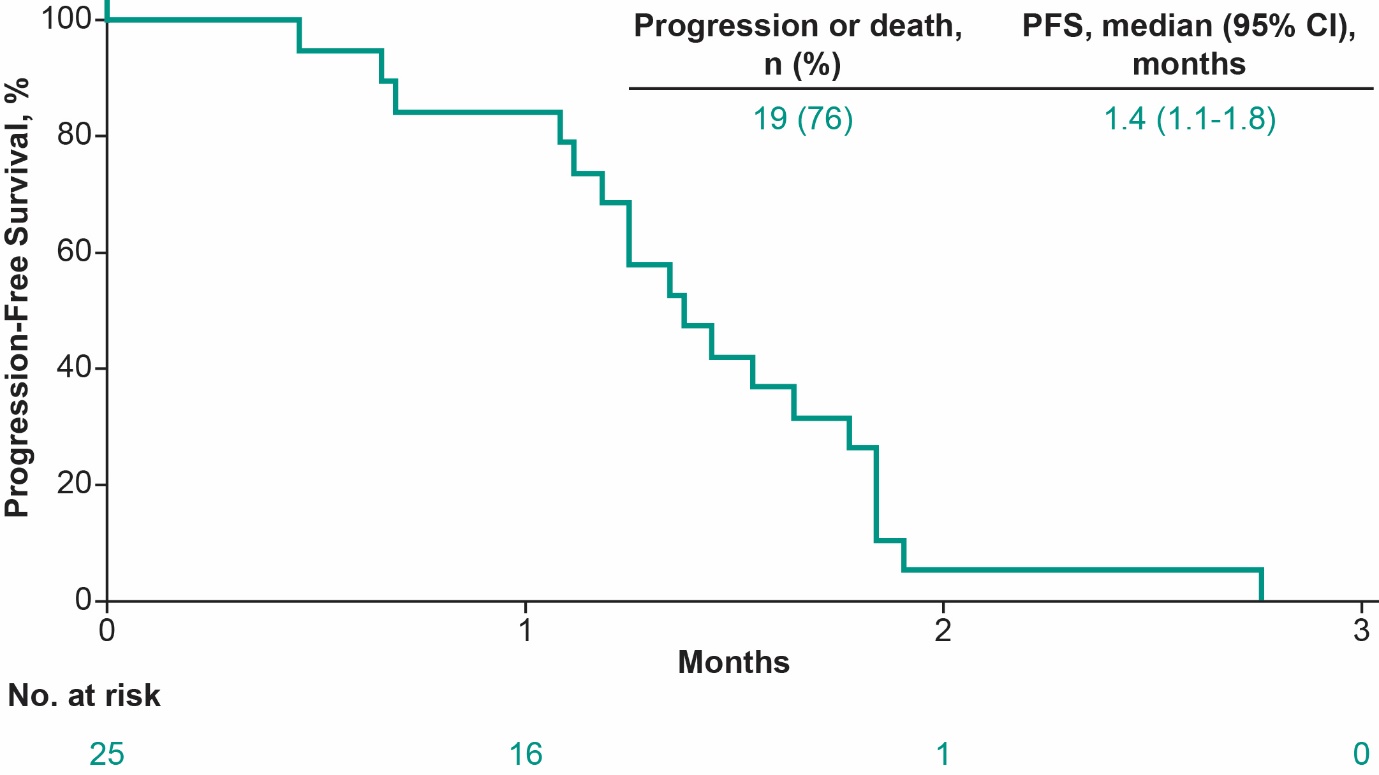


CI, confidence interval; PFS, progression-free survival.
